# One-session cognitive behavior treatment for long-term frequent attenders in primary care: randomized controlled trial

**DOI:** 10.1080/02813432.2019.1569371

**Published:** 2019-02-02

**Authors:** Sinikka Luutonen, Anne Santalahti, Mia Mäkinen, Tero Vahlberg, Päivi Rautava

**Affiliations:** a Department of Psychiatry, University of Turku, Turku, Finland;; b Turku University Hospital, Turku, Finland;; c Health and social care of Ylöjärvi, Ylöjärvi, Finland;; d Welfare Division, City of Turku, Finland;; e Department of Biostatistics, Faculty of Medicine, University of Turku, Turku, Finland;; f Department of Public Health, Faculty of Medicine, University of Turku, Turku, Finland;; g Turku Clinical Research Centre, Turku University Hospital, Turku, Finland

## Abstract

**Aim:** The aim of the study was to find out, if a single cognitive behavior treatment (CBT) session for long-term frequent attenders in primary care affects the attendance frequency and mental well-being of the patients.

**Methods:** Out of 193 long-term frequent attenders, 56 participated and were randomized to receive either a one-session CBT intervention or usual care. The groups were compared to each other regarding change in general practitioner visits and change in depressive symptoms, sense of coherence, somatoform symptoms and hypochondriacal anxiety at six months’ follow-up.

**Results:** The attendance frequency decreased in both groups, but there was no difference between the groups. Changes in mental functioning did not differ between the groups. When patients with no mental health disorder were analyzed separately, the decrease in GP visits was significantly higher in the intervention group than in the control group (*p* = .004).

**Conclusion:** A single session of CBT is not useful in reducing GP visits or improving mental well-being of long-term frequent attenders. Frequent attenders without a psychiatric disorder may benefit from this kind of intervention.

## Introduction

Primary care physicians use a disproportionate amount of time treating a relatively small number of patients who attend their practice frequently. According to Smits et al. [1], primary care physicians spend almost 40% of their time on 10% of their patients. There is no universal definition of frequent attendance; studies define frequent attenders (FA) either as the highest decile of the most frequently attending patients per gender and age group [1,2] or, especially in Finnish studies, a FA is defined as having 8–11 patient-initiated general practitioner (GP) contacts per year [3,4]. Naturally, there are situations when a person needs frequent help from the health care system. Studies have indicated that FA is often temporary and when the underlying health problem has been solved, the use of health care services diminishes, but 15% of one-year FAs continue to attend frequently in the following years [5]. Persistent FA in primary care is associated with poor quality of life and physical and mental multimorbidity [6].

Apparently, some FA patients, and especially some persistent FA patients, do not receive the help they need from the health care system. A recent review [7] showed that consistent evidence on the effects of particular interventions in specific FA patient domains is lacking. Somatoform disorders [8] and medically unexplained symptoms [9] are associated with high health care utilization, and cognitive behavior treatment (CBT) has been found effective in treating them [10–12]. Further, the efficacy of CBT for FAs has been studied with promising results [13–15].

In the present RCT, we have studied whether one single CBT session changes the attendance frequency and mental well-being of long-term FAs.

## Material and methods

The study protocol was approved by the Ethics Committee of the Hospital District of Southwest Finland (1347180/2008). The study was not registered because at the time the study was done (2009), registration of clinical trials was not a standard practice in Finland.

### Patients

The study was performed in 2009 in the city of Turku, where the entire population (178,000) has access to public health care center services. Long-term FA was defined as having at least 10 GP visits in 2008 and, in addition, at least 10 GP visits within any of the preceding three years.

The study group was identified from the databases of the Turku health care center.

**Figure 1.**
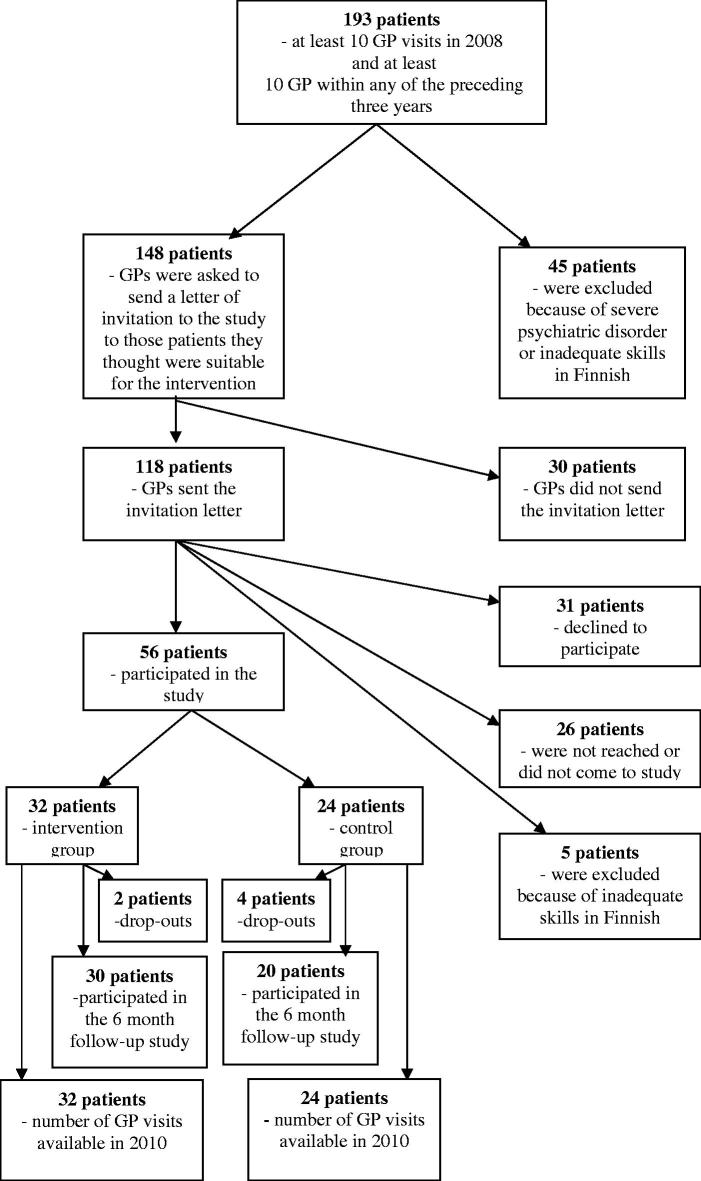
Flow chart of the study.

There were altogether 193 long-term FAs aged 18–75 years (Figure 1). Forty-five patients were excluded because of severe mental disorder, cognitive impairment or inadequate skills in Finnish. A stratified randomization by age group (≥50 years and ˂50 years) and gender was done by a statistician (TV) to form the intervention group and the control group. At this point, the allocation was concealed from other members of the research group. The GPs of the patients were contacted and asked to send a standard invitation letter to those patients they would find suitable for the intervention. This letter introduced the study method, emphasizing that the aim of the study was how to better serve the patients who needed frequent appointments. In 30 cases, no letter was sent (4 due to non-suitability, 26 cases due to non-compliance of the GPs). A research nurse contacted the patients by phone and checked the patient’s final suitability for the study. Altogether 56 patients, 32 in the intervention group and 24 in the control group, participated in the study. In comparison with the 92 patients who did not take part in the study, the participants were older (52.5 ± SD 16.8 years vs. 45.1 ± SD 16.0 years, *p* = .008) and the proportion of women was higher (83.% vs. 67.4%, *p* = .027). When receiving the time and place for the baseline interview with the research nurse, the patients were told, if they had been allocated to the intervention group or control group. The GPs were not informed, whether a patient took part in the intervention group or control group. . Members of the research group were aware of the allocation at the point of analyzing the results. Apart from the intervention, all the patients were treated as usual. All participants gave informed consent. Six patients (10.7%) were not reached at the 6-month follow-up visit, at which point they were censored. However, since we had the permission of these patients to use their patient records, all 56 patients were included when analyzing the GP visit data.

Sample size calculations were not performed before the study. However, retrospectively it was calculated that a sample size of 56 patients was sufficient to detect a mean difference of 2.9 (using of SD 3.6) between the groups in the change of GP visits with 80% power and the two-tailed 0.05 level of alfa. A difference of three visits can be considered clinically significant.

### Instruments

Depressive symptoms were measured by the 21-item Beck Depression Inventory (BDI) [16]. The sense of coherence (SOC) reflecting a person’s view of life and his/her capacity to respond to stressful situations was assessed by the Orientation to Life Questionnaire (SOC-13) [17]. Somatization was measured by the 12-item somatization subscale (SCL-SOM) of the Symptom Check List 90 (SCL-90) [18]. The Whiteley Index (WI) [19] was used to measure hypochondriacal anxiety and concern. Psychiatric diagnoses according to the Diagnostic and Statistical Manual of Mental Disorders, 4th Edition (DSM-IV) were established using the Mini International Neuropsychiatric Interview (MINI) [20]. Since the MINI lacks items to check for somatoform disorders, the somatoform section of the Structured Clinical Interview for DSM [21] was used.

### Procedure

The primary outcome of the study was the change in GP visits between 2008 and 2010. The number of GP visits was recorded from the electronic patient records. Changes in depressive symptoms, sense of coherence, somatization, and hypochondriacal anxiety were secondary outcomes. All study patients were mailed the BDI, SOC-13, SCL-SOM and WI, which they filled in at home and took with them to the baseline visit with the research nurse. Medications and disorders diagnosed by a physician were recorded. The nurse, trained and experienced in using these structured instruments, performed the MINI and somatoform section of the SCID. After six months, depressive symptoms, sense of coherence, somatoform symptoms and hypochondriacal anxiety were assessed in same way as at baseline. The number of GP visits by the study patients during 2008 and during 2010 were recorded from the electronic patient records.

### Intervention

All CBT interventions were performed by a resident in psychiatry (MM) who had no formal psychotherapy education, but had attended some CBT workshops as part of the training program for residents in psychiatry. Each CBT session took 60–90 min. During the session, the patient was encouraged to talk about the health problems that had been the reason for the GP visits. Open questions were used to allow the patient space and a feeling that the therapist was interested in the patient’s problems. Along the session, an important principle was to see the patient as the best expert of his/her health. Family, employment, social situation and special stressful or important life events during the recent years were discussed. Psychoeducation on the meaning of stress for well-being and bodily sensations was given and methods to release stress symptoms with physical activity were presented and screened against the patient’s specific situation giving the patient leadership. The importance of cognitions in amplifying somatic sensations was explained on a general level and then applied to the patient’s individual experience. The patient’s recent situations with somatic symptoms were used to demonstrate the link between thoughts, affect, behavior, and biology (cognitive conseptualisation). The goal was to help the patient to identify dysfunctional thoughts with a negative effect on symptoms and to help him/her to find new more adaptive and less catastrophizing thoughts related to the situation and symptoms (cognitive reattribution). The patient was offered a short leaflet about stress, well-being and measures to release these symptoms. At the end of the session, a more useful way of using health care services (e.g., always visiting the same GP, asking the GP for regular pre-determined appointments instead of using emergency visits) was discussed. The patient was also asked how health care services should be offered to better meet the needs.

### Statistics

Data were analyzed using IBM SPSS Statistics for Windows (version 21.0, IBM Corp., Armonk, NY, USA). Pearson’s *χ*^2^-test or Fisher’s exact test was used for categorical variables and the two-sample T-test or Mann-Whitney’s U-test for continuous variables to compare patient characteristics between intervention and control groups. The paired T-test or Wilcoxon’s signed rank test was used to compare the change from baseline to follow-up within groups. The changes in the BDI, SOC and SCL-SOM scores between intervention and control groups were compared by the two-sample T-test or Mann-Whitney’s U-test to analyze the effect of the intervention. P-values below 0.05 were considered statistically significant.

## Results

Of all the patients, 91.1% had a somatic diagnosis, musculoskeletal disorders being the most prevalent (51.8%). The mean number of somatic diagnoses was 1.95 (SD 1.30). The intervention and the control groups did not differ from each other regarding somatic morbidity and the number of medications. Of the patients, 41% had at least one psychiatric disorder. Patients in the control group had a somatoform disorder more often than patients in the intervention group (*p* = .035). Regarding other psychiatric diagnoses there were no differences between the groups. Patients in the control group lived more often in a couple relationship than those in the intervention group (*p* = .017). Otherwise there were no sociodemographic differences between the groups. The baseline BDI, SOC-13, SCL-SOM and WI scores or GP visits in 2008 did not differ between the groups. (Table 1)

**Table 1.** Patient characteristics.

Variable	All	Intervention group	Control group	*p*
	*n* = 56	*n* = 32	*n* = 24	
Age Mean (SD)	52.5 (16.8)	52.9 (17.5)	52.2 (16.3)	ns
Female n (%)	47 (83.9)	26 (81.3)	21 (87.5)	ns
Living in a couple relationship n (%)	27 (48.2)	11 (34.4)	16 (66.7)	<.05
Unemployed n (%)	2 (3.6)	1 (3.1)	1 (4.2)	ns
Unable to work because of chronic illness n (%)	17 (30.4)	8 (25.0)	9 (37.5)	ns
Years of education median (IQR)	12.0 (6.0)	12.0 (6.0)	12.0 (5.0)	ns
Depressive disorder n (%)	15 (26.8)	8 (25)	7(29.2)	ns
Anxiety disorder n (%)	16 (28.6)	10 (31.3)	6 (25.0)	ns
Substance use disorder n (%)	8 (14.3)	3 (9.4)	5 (20.8)	ns
Psychotic disorder n (%)	5 (8.9)	3 (9.4)	2 (8.3)	ns
Somatoform disorder n (%)	7 (12.5)	1 (3.1)	6 (25.0)	< .05
Any mental disorder n (%)	23 (41.1)	12 (37.5)	11 (45.8)	ns
Cardiovascular disorder n (%)	21 (37.5)	11 (34.4)	10 (41.7)	ns
Musculoskeletal disorder n (%)	29 (51.8)	16 (50.0)	13 (54.2)	ns
Pulmonary disorder n (%)	19 (33.9)	12 (37.5)	7 (29.2)	ns
Neurological disorder n (%)	14 (25.0)	5 (15.6)	9 (37.5)	ns
Endocrine disorder n (%)	12 (21.4)	7 (21.9)	5 (20.8)	ns
Gastrointestinal disorder n (%)	10 (17.9)	6 (18.8)	4 (16.7)	ns
Cancer n (%)	4 (7.1)	3 (9.4)	1 (4.2)	ns
Any somatic disorder n (%)	50 (89.3)	28 (87.5)	22 (91.7)	ns
Number of regularly used medications Median (IQR)	4.0 (5.0)	5.0 (5.0)	3.0 (6.75)	ns
BDI (baseline) Mean (SD)	13.0 (10.1)	12.4 (10.5)	13.7 (9.7)	ns
SOC-13 (baseline) Mean (SD)	59.3 (12.5)	59.7 (12.3)	58.7 (13.0)	ns
SCL-SOM (baseline) mean (SD)	5.5 (3.0)	5.5 (3.2)	5.1 (2.8)	ns
WI (baseline) Median (IQR)	4.5 (5.0)	6.0 (7.0)	4.0 (5.0)	ns
GP visits in the year before the study Median (IQR)	11.0 (4.0)	11.0 (5.0)	11.0 (2.0)	ns

Medians are shown if the variable is non-normally distributed.

BDI: Beck Depression Inventory; SOC-13: Orientation to Life Questionnaire; SCL-SOM: somatization subscale of the Symptom Check List 90; WI: Whiteley Index; GP: general practitioner; SD: standard deviation; IQR: interquartile range.

Six patients did not participate in the 6-month follow-up. The drop-outs did not differ from the other patients regarding baseline BDI, SOC-13, SCL-SOM and WI scores or GP visits in 2008.

The number of GP visits decreased significantly both in the intervention group (*p* < .001) and in the control group (*p* = .001) during the study. There was a significant decrease in BDI scores in the intervention group (*p* = .024) but not in the control group. SOC-13 scores increased significantly in the control group (*p* = .043), but not in the intervention group. The SCL-SOM and WI scores did not change within the groups during follow-up (Table 2).

**Table 2.** Change in Beck Depression Inventory (BDI), Orientation to Life Questionnaire (SOC-13), somatization subscale of the Symptom Check List 90 (SCL-SOM), Whiteley Index (WI) and GP visits at baseline (BL) and follow-up (F-U). For GP visits, the baseline score is the number of visits in 2008 and the follow-up score is the number of visits in 2010.

Variable	Intervention group				Control group				P Between groups (regarding change)
	BL	F-U	Change between BL and F-U	P within group	BL	F-U	Change between BL and FU	P within group	
BDI				< .05				ns	ns
Mean	12.4	10.8	−2.3		13.7	12.6	−1.0		
SD *n* = 49	10.5	10.6	5.1		9.7	11.1	4.0		
SOC-13				ns				< .05	ns
Mean	59.5	60.7	1.1		58.6	63.1	4.5		
SD *n* = 49	12.9	12.4	8.5		13.4	11.7	9.2		
SCL-SOM				ns				ns	ns
Mean	5.6	5.2	−0.4		4.9	4.6	−0.3		
SD *n* = 47	3.2	2.9	2.4		2.7	3.1	2.6		
WI				ns				ns	ns
Median	6.0	3.5	0.0		4.0	4.5	1.0		
IQR *n* = 45	7.0	7.25	3.0		5.0	4.75	3.0		
GP visits				< .001				< .05	ns
Median	11.0	7.0	−5.0		11.0	7.5	−4.0		
IQR *n* = 56	5.0	6.5	5.0		2.0	6.75	3.75		

For the other variables, baseline is the first interview followed by the 6-month follow-up.

SD: standard deviation; IQR: interquartile range.

The decrease in GP visits was similar in both groups, nor did the changes in BDI, SOC-13, SCL-SOM and WI scores between the intervention and the control group differ from each other during follow-up (Table 2).

When patients with no mental health disorder (*n* = 33) were separately analyzed, the change in GP visits was significantly bigger in the intervention group than in the control group (median −5.5, interquartile range IQR 4.0 vs. median −3.0, IQR 3.5, *p* = .004). In the group of patients with at least one mental health disorder (*n* = 23), the difference in GP visit changes between the intervention group (median −3.0, IQR 4.0) and the control group (median −5.0, IQR 5.0) was not significant (*p* = .128).

## Discussion

In our study, a single CBT session for long-term FAs did not have an effect on attendance frequency or mental well-being at follow-up six months later. However, when FAs with no psychiatric disorders were separately considered, there was a significant difference in attendance frequency between the intervention and the control group favoring the intervention.

Earlier, the effect of CBT interventions on attendance frequency and mental well-being has mainly been studied in patients with somatoform disorder or medically unexplained symptoms [11,12] or FAs with medically unexplained symptoms [13,14]. Sumathipala et al. [11] and Martin et al. [12] reported reduced attendance frequency and improved mental well-being, van Ravesteijn et al. [13] improved mental well-being and Baker et al. [14] reduced attendance frequency. In our study, only 13% of participants had a somatoform disorder. Possibly, FAs with somatoform disorders or medically unexplained symptoms are better candidates for CBT than FAs in general. To our knowledge, the study by Malins et al. [15] has been so far the only CBT study dealing with FAs without the inclusion criteria of somatoform disorders or medically unexplained symptoms. They showed a reduction in health care services and an improvement in mental health outcomes. However, they did not have a control group.

Our goal was to find out if a one-session CBT is an easy and quick method to help long-term FAs. It is noteworthy that only 56 out of 148 FAs (38%) took part in the study. We found that although these patients consulted physicians on their own initiative, it was difficult to reach them or they preferred to decline participation in the study. In the CBT study by Malins et al. [15], the proportion of patients participating in the intervention study was even lower: only 19% of long-term FAs agreed to attend a baseline assessment and only 7% were offered and accepted CBT. They conclude that CBT is feasible and acceptable only for a small subgroup of long-term FAs. In our study, the intervention consisted of only one session, which might be more acceptable to FA-patients than an intervention including several sessions. On the other hand, maybe one 60–90 min session is not enough. In the study by Sumathipala et al. [11], there were 6 × 30 min sessions, in the study by van Ravasteijn et al. [13], there were 8 group sessions and in the study by Malins et al. [15] the median number of sessions was 11. Martin et al. [12] found one-session group CBT useful. In their study, the group session took 3 – 4 h.

When only patients without a psychiatric disorder were considered in our study, there was a significant difference in the reduction of attendance frequency favoring the intervention group. Of our patients, 41% had at least one psychiatric diagnosis. In the earlier CBT intervention studies, the proportion of patients with a psychiatric diagnosis has been higher [12,15] or has not been reported [11,13], and patients with no psychiatric disorder have not been analyzed separately. A single 60–90 min session with elements of psychoeducation, cognitive restructuring, and provision of information about health care services may be sufficient to help a mentally healthy person to use health care services in a more adequate and helpful way, but the group of mentally healthy patients was small and this conclusion may be premature.

To our knowledge, our study is the first RCT study assessing the usefulness of CBT in treating long-term FAs without the inclusion criterion of medically unexplained symptoms. A strength of this study is that we had possibility to reach all adult (18–75 years) primary care patients who had been FAs during two years in a given area. The small number of FA patients who ultimately participated in study and a relatively short follow-up time are limitations of the study. It is possible that after refusals, exclusions, and drop-outs, the sample size was too small to detect a difference in visit numbers. On the other hand, the small number of participators may indicate that this kind of psychosocial intervention is feasible only to a subgroup of FAs. Selection of participants was made by GPs causing a possible selection bias. Our intention was to use the expertise of GPs to exclude those of their patients for whom they felt the intervention was not suitable. However, most of the exclusion at this point was due to forgetfulness of the GPs, which, on the other hand, may illustrate the workload of the GPs. We do not have the information, if the patients were treated by the same GP in 2008 and 2010; lack of continuity of care may affect attendance frequency. Further, we do not have data on the patients’ sick leave days before and after the intervention, so we could not assess the effect of the intervention on work ability.

In conclusion, one-session CBT was not useful in reducing GP visits or improving mental functioning in long-term FAs. The subgroup of FA patients without a psychiatric diagnosis seem to benefit from the intervention, but further studies are needed to confirm this finding.
